# Radio-Oxidation Ageing of XLPE Containing Different Additives and Filler: Effect on the Gases Emission and Consumption

**DOI:** 10.3390/polym13172845

**Published:** 2021-08-24

**Authors:** Muriel Ferry, Floriane Carpentier, Manon Cornaton

**Affiliations:** Université Paris-Saclay, CEA, Service d’Etude du Comportement des Radionucléides, 91191 Gif-sur-Yvette, France; floriane.carpentier@cea.fr (F.C.); manon.cornaton@cea.fr (M.C.)

**Keywords:** formulated polyethylene, γ irradiation, radio-oxidation, antioxidants, flame retardant, dose effect, dose rate effect

## Abstract

In the lifetime extension of nuclear power plants (NPPs) context, aging of electric cables has to be very well understood in order to predict their end-of-life and thus to replace them on time. Therefore, evaluation and understanding of the ageing mechanism of the cable insulating material is mandatory under conditions as close as possible of those encountered in NPPs. In this context, different formulated crosslinked polyethylenes (XLPE)—one of the polymers used nowadays to manufacture the insulator layer—have been irradiated under oxidative conditions, at two different dose rates and at different aging doses. Gases emitted and consumed from the irradiated polymers were quantified to identify the primary processes happening in the materials and thus the interactions involved between the different molecules composing the formulated polymers.

## 1. Introduction

The lifetime extension of nuclear power plants (NPPs) from 40 to 60 years is currently under study in France [1]. In this context, the aging of all the safety components has to be evaluated to predict when such components will have to be replaced. Speaking about nuclear materials often refers to metals and alloys, but polymers represent also safety organs of primary importance, as they are part of electric cables. NPPs contain thousands of kilometers of these cables, of any type and size. Since the construction of the first NPP, very different polymers were used, including halogenated polymers as Neoprene^®^ and PVC (polyvinylchloride) [2,3,4,5,6,7,8], aliphatic polymers, such as PE (polyethylene) and EPR (ethylene/propylene copolymers) [9,10,11,12,13,14,15,16], or relatively more recently EVA/EPDM (ethylene/vinyl acetate copolymers crosslinked with ethylene/propylene/diene terpolymer) [17,18,19,20]. Nowadays, one of the main polymers employed to manufacture cables is XLPE (crosslinked polyethylene).

Whatever its use, a polymer is rarely employed pure. To improve some specific properties and/or to increase their lifetime, additives and fillers are added to the formulation. As examples, phthalates are added to improve the plasticity of polymers—of PVC in most cases [21]—whereas aluminum trihydrate is added as a flame retardant [22,23]. The most known class of additives is the antioxidants one, because these molecules significantly increase polymer service life, and are even added in a relatively small proportion—generally about 1 phr (phr means parts per hundred rubber: 10 phr of additive means 10 g of additive have been added to 100 g of polymer). All these added molecules modify polymer aging because of the interaction with the unstable species generated in the polymer during aging or simply because of a “dilution” effect, i.e., a less important part of organic matter in the formulated material [8,18,24,25,26,27,28,29]. 

In the NPP service life extension context, one concern is the aging of these organic materials and their lifetime prediction. Among the aging factors encountered are the surrounding temperature, the dose rate, the total dose, the presence of oxygen, the relative humidity, the presence of ozone, and/or of chemical contaminant. In practice and in the reactor building where the most penalizing conditions are met, the aging factors to take into account are a temperature of about 50 °C, a dose rate of 0.1 Gy·h^−1^, and the presence of oxygen. To be as representative as possible of these conditions, accelerated irradiations have to be implemented, but with conditions that need to be carefully evaluated. As an example, in thermo-oxidation aging conditions, Bartoníček et al. [30] recommend not to exceed the nominal temperature by more than 20 °C to maintain reliable thermo-oxidation conditions. 

The objective of this article is to understand the aging mechanism of formulated crosslinked polyethylene upon radio-oxidation; these materials being more and more commonly employed as cable insulators. To perform this study, formulated model XLPEs with increasing quantities of different added additives and fillers were irradiated under oxidative conditions at two different dose rates and at different doses. Gases release during irradiation gave information on primary processes that modify the polymers at different ages: we decided to focus on these kinds of modifications. Hence, the emission of different gases, along with oxygen consumption, were evaluated depending on the formulated material, the dose rate, and the dose. This work allowed obtaining a better understanding of the interactions involved between the different molecules composing the formulated polymer; the final goal was to be able, in the future, to give a better lifetime prediction for electric cables in NPPs. 

## 2. Materials and Methods

### 2.1. Materials

Synthesis of the crosslinked polyethylene XLPE was performed by reacting linear low-density polyethylene LLDPE with vinyltrimethoxysilane as a crosslinking agent and dicumyl peroxide as an initiator. The LLDPE chosen for this study presents a density of 0.918 g·cm^−3^ and a melting point of 120 °C. This polymer contains a very small quantity of antioxidants (mainly BHT and Irganox 1076, according to Xu et al. [31]) and was used as received without any further purification. 

**Figure 1:**
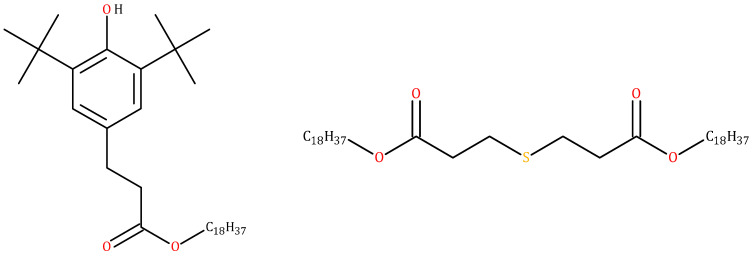
Chemical formulae of Irganox 1076, on the left, and of Irganox PS802, on the right.

Two antioxidants were used is this work. The primary antioxidant chosen was Irganox 1076 (generic name: octadecyl 3-(3,5-di-tert-butyl-4-hydroxyphenyl)propionate); its chemical formula is given Figure 1 (left). The secondary antioxidant chosen for this work was Irganox PS802 (generic name: distearyl thiodipropionate); its chemical formula is given Figure 1 (right). One flame retardant, ATH, was also added to some samples. It corresponded to aluminum trihydrate, of which the chemical formula is Al(OH)_3_. 

Samples were furnished by Nexans France in the form of tapes about 500 µm thick. In this work, different formulations were evaluated:

- XLPE M1: XLPE “as received”
- XLPE M2(Irg1076): XLPE + 1 phr of Irganox 1076
- XLPE M3(IrgPS802): XLPE + 1 phr of Irganox PS 802
- XLPE M4(Irg1076-IrgPS802): XLPE + 1 phr of Irganox 1076 + 1 phr of Irganox PS 802
- XLPE M5(ATH25): XLPE + 25 phr of ATH
- XLPE M6(ATH50): XLPE + 50 phr of ATH
- XLPE M7(Irg1076-IrgPS802-ATH50): XLPE + 1 phr of Irganox 1076 + 1 phr of Irganox PS 802 + 50 phr of ATH

### 2.2. Irradiation Conditions

Accelerated aging irradiations were performed at the Panoza facility (UJV, Rez, Czech Republic), using a ^60^Co source. Alanine dosimeters were used, without further electronic correction, to take into account the electronic density difference between water and polymers. Uncertainties in given doses were about 6.5%. Formulated polymer tapes were placed at a given and known distance from the source to obtain the desired dose rate. Air flowed inside the irradiator chamber, and the temperature was roughly constant and approximately equal to 45 °C.

In this experimental series, two dose rates were employed, and for each dose rate, three doses were achieved:

- Low dose rate corresponds to 5 Gy·h^−1^. The three doses achieved at this dose rate were equal to 25 kGy, 67 kGy, and 138 kGy.
- Medium dose rate corresponds to 40 Gy·h^−1^. The three doses achieved at this dose rate were equal to 67 kGy, 220 kGy, and 374 kGy.

To analyze gases emission and oxygen consumption, polymers have to be irradiated in closed containers. Hence, a second irradiation has to be performed, at doses as low as possible in comparison to the initial dose in order to keep the material signature. The use of this two-step irradiation protocol has already been presented in previous articles [32,33,34] and has many advantages. For instance, when a polymer is irradiated under an oxidative atmosphere, radicals are formed and react with oxygen—the well-known radio-oxidation. In case of irradiation in a closed container and if the dose is important, oxygen is consumed up to its depletion; in such case, irradiation is not homogeneous and results are unusable. The two-steps protocol allows to avoid such experimental drawbacks, among others.

**Figure 2:**
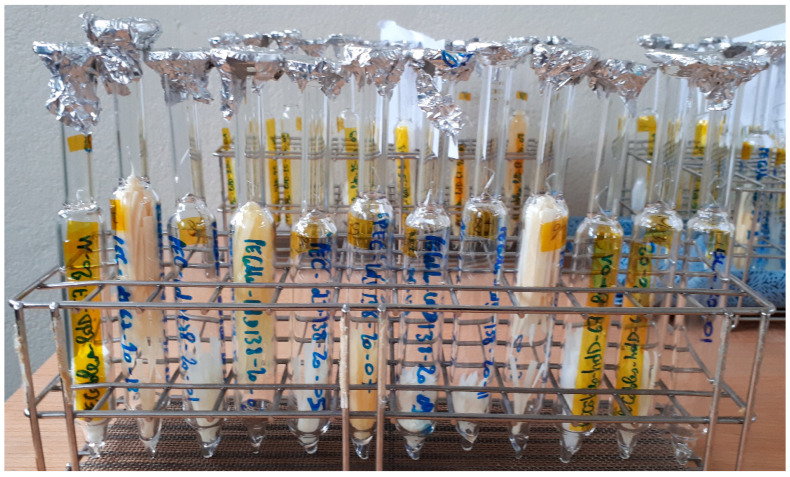
Photos of glass closed ampoules, which contain polymer.

To prepare the second step irradiation, polymers were placed in glass closed ampoules, as presented in Figure 2. The atmosphere inside the containers was introduced at a known pressure before sealing. We chose reconstituted air (20.0% O_2_, 77.99% N_2_ and 2.01% Kr). In this gas mixture, krypton was used as a tracer to determine the final pressure after irradiation inside the closed container because of its inertness towards irradiation. After preparations, the second irradiation was performed using the POSEIDON facility (LABRA, CEA Saclay, France), using a ^60^Co source. Dosimetry was performed using a UNIDOS PTW dosimeter equipped with a calibration chamber. Dosimetry was performed just before irradiation by placing the ionization chamber of the dosimeter at the same place as the samples. In these irradiation conditions as well, no electronic correction was realized. Uncertainties on given doses were less than 6%. In the present study, the dose rate achieved was of 1.06 kGy·h^−1^ and for each formulated polymer at each dose, two glass containers were irradiated at two low different doses, i.e., 12.7 and 25.4 kGy.

Between the two dose steps, samples were stored under an inert atmosphere in the dark to prevent or at least reduce further aging due to post-oxidation. 

### 2.3. Radiation Chemical Yields Determination

Gas analyses were performed using a high resolution gas mass spectrometer from Thermo Fischer Scientific (MAT-271) [34]. This mass spectrometer is equipped with a ionization chamber in which gas ionization is realized through electron impact. The gas fragments (or ions) produced by the ionization step are separated using a magnetic sector. Finally, ion detection of the different ions is performed by a Faraday cup and electron multiplier—depending on the m/z fragment concentration. The mass ranged from 1 to 100 amu. 

The instantaneous formation rate of gas X, $G_{D}\left(X\right)$ was determined using its partial pressure after irradiation in a closed container according to Equation (1), given below. This formation rate is called $G_{D}\left(X\right)$ because it is equivalent to the radiation chemical yield extrapolated at dose *D*.

(1) $$G_{D}\left(X\right)=\frac{1}{d}\cdot \frac{d\left[X\right]}{dt}=\frac{P_{f}\cdot {\%}_{vol,X}\cdot V_{free}}{R\cdot T\cdot \Delta D\cdot m}$$

where d is the dose rate in Gy·s^−1^, *[X]* is the concentration of the gas X in mol·kg^−1^ measured after irradiation at a given dose ΔD in Gy, $P_{f}$ is the total pressure in the glass ampoule at the end of the irradiation in Pa, ${\%}_{vol,X}$ is the gas X volume fraction, $V_{free}$ is the free volume in the glass ampoule in m^3^, *R* is the gas constant, *T* is the sample’s temperature under irradiation in K, and *m* is the mass of the irradiated sample in kg. 

Uncertainty was always inferior to 10% in the hydrogen emission case; it was slightly higher than 10% in case of oxygen consumption and of carbon dioxide release.

## 3. Results

### 3.1. Radiation Chemical Yields Extrapolated at Zero Dose

Table 1 summarizes the different radiation chemical yields extrapolated at zero dose. Error bars correspond either to the mass spectrometry uncertainty or to the standard deviation between the two glass ampoules of each sample, whichever was higher.

Whatever the dose rate, in case of XLPE without inorganic matter—from XLPE M1 to XLPE M4(Irg1076-IrgPS802)—the hydrogen radiation chemical yields extrapolated at zero dose correspond to the mean value obtained from the compiled data of the literature: (3.6 ± 0.4) × 10^−7^ mol·J^−1^ [35]. The hydrogen formation reaction is known to be very fast because of the extremely high reactivity of the free radicals leading to the formation of this gas. As all the fully organic materials present equivalent radiation to chemical yields extrapolated at zero dose, this implies that none of the antioxidants in this study are protecting the polymer chains from the primary reactions induced by the interaction of ionizing rays/matter. This result was attempted because the formation of hydrogen is faster than the formation of peroxide radicals and, thus, of hydroperoxydes, on which antioxidants act. Nonetheless, Irganox 1076 contains an aromatic ring, an efficient energy sink [36,37,38,39], and no effect was observed. This implied that the Irganox 1076 concentration was not sufficient to give an efficient radiation protection effect in regards to the energy transfer and primary mechanisms. 

Table 1 shows also that for XLPE M1, XLPE M3(IrgPS802), XLPE M5(ATH25), and XLPE M6(ATH50), the oxygen consumption radiation chemical yields extrapolated at zero dose were markedly higher than those for materials containing Irganox 1076, i.e., XLPE M2(Irg1076), XLPE M4(Irg1076-IrgPS802) and XLPE M7(Irg1076-IrgPS802-ATH50). On the one hand, the G_0_(-O_2_) was higher than 12 × 10^−7^ mol·J^−1^ whereas it ranged between 4 and 7 × 10^−7^ mol·J^−1^ for materials protected with the primary antioxidant. This important difference with and without Irganox 1076 proves its effective protection on the oxidation process, the primary antioxidant being very efficient in the termination of the propagation reaction of the Bolland and Gee mechanism [40]. Hence, it limits, in a very important way, the reaction of oxygen with the radicals formed in the XLPE polymers because of the γ-irradiation. This result is also confirmed by the G_0_(CO_2_) of the different materials, which were also far less important for XLPE M2(Irg1076), XLPE M4(Irg1076-IrgPS802), and XLPE M7(Irg1076-IrgPS802-ATH50) than for XLPE M1, XLPE M3(IrgPS802), XLPE M5(ATH25), and XLPE M6(ATH50). This gas is formed by decomposition of oxidized species—stable or not [41,42,43]—and these oxidized species are necessarily formed by mechanisms that require prior oxygen incorporation in the polymer chain. The less oxygen incorporated in the polymer, the less carbon dioxide release possible. 

The effect of the presence of the secondary antioxidant Irganox PS802 could be evaluated by comparing XLPE M1 and XLPE M3(IrgPS802) on one hand and XLPE M2(Irg1076) and XLPE M4(Irg1076-IrgPS802) on the other. As there is no observable difference between these two couples, it can be deduced that the secondary antioxidant Irganox PS802 has no effect on the gas release and consumption, neither alone nor in combination with the primary antioxidant. This can be explained by the fact that secondary antioxidants decompose hydroperoxides to stabilize aged materials [44]. At the very low doses employed to evaluate the radiation chemical yields extrapolated at zero dose, it can probably be assumed that the concentration of hydroperoxides was not high enough to initiate their decomposition. 

In case of XLPE materials which contain inorganic matter—from XLPE M5(ATH25) to XLPE M7(Irg1076-IrgPS802-ATH50)—the hydrogen radiation chemical yields extrapolated at zero dose were noticeably lower than the mean value in the literature [35]. Nonetheless, by normalizing the radiation chemical yields obtained to the organic content of the materials, the mean value of the literature was retrieved. In a concomitant way, normalizing the oxygen consumption and the carbon dioxide emission radiation chemical yields extrapolated to zero dose by the organic content in the XLPE under study allowed to retrieve the value of XLPE M1 as well. This implies that, at least at very low doses, the flame retardant presence in the polymer had no effect on the degradation mechanisms induced by the irradiation under oxidative atmosphere in the XLPE, at the molecular level. 

### 3.2. Dose Effect

**Figure 3:**
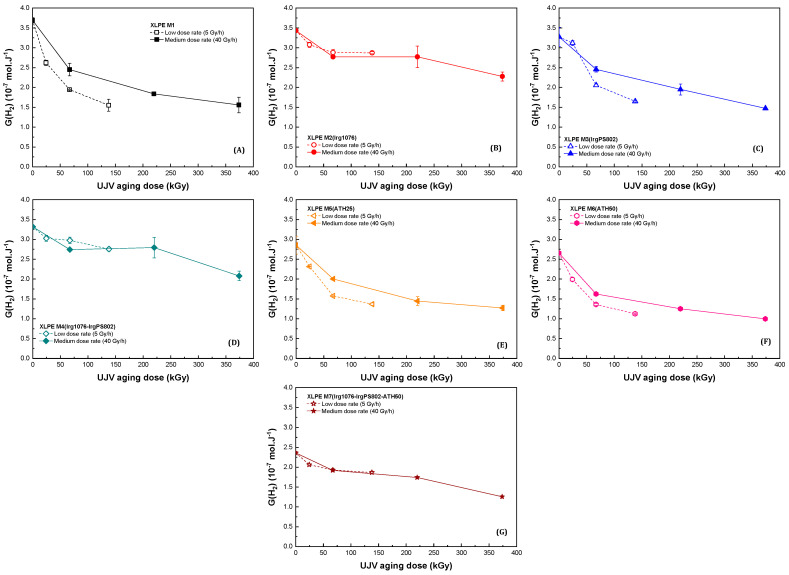
Hydrogen emission radiation chemical yield evolutions as a function of dose at two different dose rates (open symbols for 5 Gy·h^−1^ and solid symbols for 40 Gy·h^−1^), for (**A**) XLPE M1, (**B**) XLPE M2(Irg1076), (**C**) XLPE M3(IrgPS802), (**D**) XLPE M4(Irg1076-IrgPS802), (**E**) XLPE M5(ATH25), (**F**) XLPE M6(ATH50), and (**G**) XLPE M7(Irg1076-IrgPS802-ATH50).

Figure 3 presents the hydrogen emission radiation chemical yields obtained for the seven different materials in this study.

Whatever the XLPE polyethylene under study, the hydrogen radiation chemical yield decreases when the dose increased. This dose effect has previously been observed under an inert atmosphere [45,46] as well as under an oxidative atmosphere [32] when pure polyethylene was irradiated. The observed decrease has been previously ascribed to the formation of new bonds, also called defects, which act as energy scavengers [45,46]. This behavior implies that under homogeneous irradiation conditions, the higher the defect concentrations, the lower the G_D_(H_2_). We can define a hydrogen release decrease factor as the ratio between the non-irradiated material to the same material irradiated at the highest dose. This factor is determined to be roughly equal to 2.3 for XLPE M1, XLPE M3(IrgPS802), XLPE M5(ATH25), and XLPE M6(ATH50), and to 1.4 for XLPE M2(Irg1076), XLPE M4(Irg1076-IrgPS802), and XLPE M7(Irg1076-IrgPS802-ATH50)7. As already introduced in the Section 3.1, the higher the defect concentrations, the higher the hydrogen radiation chemical decrease. Hence, this decrease factor is indirectly proportional to the quantity of defects. It can be deduced that the quantity of defects which behave as energy sink and which are formed by the radiation oxidation process in XLPE M1, XLPE M3(IrgPS802), XLPE M5(ATH25), and XLPE M6(ATH50) is noticeably higher than in XLPE M2(Irg1076), XLPE M4(Irg1076-IrgPS802) and XLPE M7(Irg1076-IrgPS802-ATH50). The difference between these two groups of XLPE being the presence of Irganox 1076 in the second group, it can be deduced that this primary antioxidant is conferring an important protection to radio-oxidation—pointing out, even indirectly, its efficiency. 

**Figure 4:**
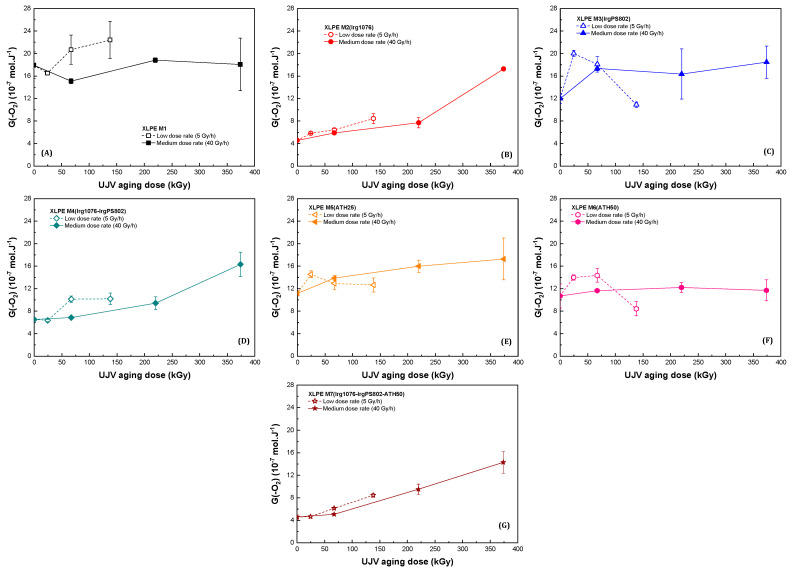
Oxygen consumption radiation chemical yields evolution as a function of dose at two different dose rates (open symbols for 5 Gy·h^−1^ and solid symbols for 40 Gy·h^−1^), for (**A**) XLPE M1, (**B**) XLPE M2(Irg1076), (**C**) XLPE M3(IrgPS802), (**D**) XLPE M4(Irg1076-IrgPS802), (**E**) XLPE M5(ATH25), (**F**) XLPE M6(ATH50), and (**G**) XLPE M7(Irg1076-IrgPS802-ATH50).

**Figure 5:**
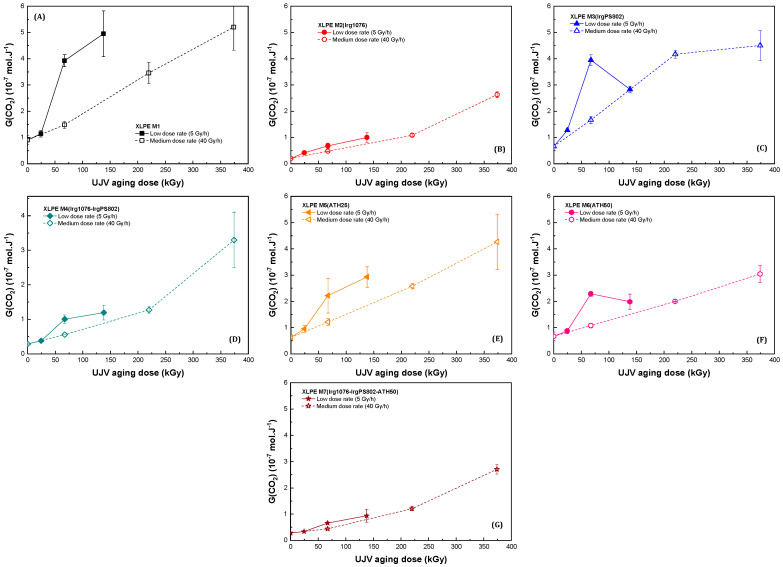
Carbon dioxide emission radiation chemical yields evolution as a function of dose at two different dose rates (open symbols for 5 Gy·h^−1^ and solid symbols for 40 Gy·h^−1^), for (**A**) XLPE M1, (**B**) XLPE M2(Irg1076), (**C**) XLPE M3(IrgPS802), (**D**) XLPE M4(Irg1076-IrgPS802), (**E**) XLPE M5(ATH25), (**F**) XLPE M6(ATH50), and (**G**) XLPE M7(Irg1076-IrgPS802-ATH50).

Figure 4 and Figure 5 present, respectively, the oxygen consumption and the carbon dioxide release radiation chemical yields obtained for the seven different materials of this study. 

Figure 4 and Figure 5 highlight the effect of the presence of Irganox 1076 already observed in the case of the radiation chemical yields extrapolated at zero dose. In the case of the XLPE materials without hindered phenol molecules, i.e., for XLPE M1, XLPE M3(IrgPS802), XLPE M5(ATH25) and XLPE M6(ATH50), the radiation chemical yields of oxygen consumption are roughly constant with dose in the dose range studied. Oxygen reacting with radicals formed in the polymer, this constant evolution can be explained by a competition between two mechanisms acting on radical formation when dose increases. First, there is a decrease in the radical formation because of the quantity of defects present in the polymer that increases with dose, these new bonds acting as energy sink (cf. just above). Second, there is an increase in the radical formation because of the degradation of these energy sinks, which form new radicals. The increase in the oxidized defects degradation is highlighted by the increase of carbon dioxide release (Figure 5), this gas being formed by decomposition of oxidized species. In the case of XLPE M2(Irg1076), XLPE M4(Irg1076-IrgPS802), and XLPE M7(Irg1076-IrgPS802-ATH50) at a medium dose rate, Figure 4 and Figure 5 show that there is a progressive acceleration in the formation of G_D_(-O_2_) and G_D_(CO_2_) with the dose irradiated. This acceleration probably comes from the Irganox 1076 consumption upon irradiation, the shape of the oxygen consumption as a function of dose having already been observed and explained in such a way [47].

The effect of the presence of the secondary antioxidant Irganox PS802 can be evaluated, as for materials irradiated at very low dose (cf. Section 3.1) by comparing XLPE M1 and XLPE M3(IrgPS802) on the one hand and XLPE M2(Irg1076) and XLPE M4(Irg1076-IrgPS802) on the other. It can be observed that whatever the dose, in the dose range of this study, there was no observable difference between these two couples. Hence, the secondary antioxidant Irganox PS802 has no effect on the gases released and consumed, neither alone nor in combination with the primary antioxidant. This result is more surprising than at a very low dose, because at the high dose employed in this study, hydroperoxides should be at a non-negligible concentration. We will come back on this point in the Discussion section.

In the same way as in the case of Irganox PS802, whatever the dose, the presence of the flame retardant ATH did not lead to any differences in gases emissions and consumption radiation chemical yields, after normalization to the organic content in the material. Hence, the absence of the effect of the presence of this inorganic molecule observed by comparing the different materials at zero dose, at the molecular scale, was retrieved whatever the dose—in the dose range of this study.

### 3.3. Dose Rate Effect

Figure 3, Figure 4 and Figure 5 present the dose effect and also the dose rate effect. They all show that there was a dose rate effect on the hydrogen emission but only in the cases of XLPE M1, XLPE M3(IrgPS802), XLPE M5(ATH25), and XLPE M6(ATH50), i.e., for materials that do not contain the primary antioxidant.

Equivalent to the observations realized in the case of the analysis of the dose effect, this difference is ascribed to a difference in the defect concentration. For these materials, it is supposed that the more important G_D_(H_2_) decrease is an illustration of the Decker and Mayo relationship between oxidation of a material and the dose rate [47]: the lower the dose rate, the higher the quantity of oxidized defects in the material. This assumption is validated by the observation of the carbon dioxide emission, this gas being necessarily formed by mechanism(s) that require prior oxygen incorporation in the polymer chain.

It has to be pointed out that in case of XLPE M3(IrgPS802) and XLPE M6(ATH50) at low dose rate, the evolutions of G_D_(-O_2_) and of G_D_(CO_2_) are not so monotonic; there is a decrease of both radiation chemical yields at 138 kGy. We believe that these data were different from the others because of a quantity of crosslinks that is different for these samples than for the others or because of a slightly lower oxygen supply during the irradiation for these samples than for the others (this because all the samples were irradiated at the same time, in a constrained volume). We think that the observed decrease is not representative of a real oxidation depletion: radiation chemical yields evolutions have to be evaluated in these two cases as a trend. 

In presence of Irganox 1076, i.e., in case of XLPE M2(Irg1076), XLPE M4(Irg1076-IrgPS802), and XLPE M7(Irg1076-IrgPS802-ATH50), the hydrogen and carbon dioxide emissions as well as the oxygen consumption are equivalent at both dose rates. As hydrogen decrease is linked to the quantity of new bonds in polymer chains, it is deduced that for materials containing the primary antioxidant, the quantity of defects is the same at both dose rates. This assumption is also supported by the CO_2_ emission concentrations, that were equivalent for the two dose rates. Hence, no dose rate effect was observed for materials containing Irganox 1076. We will come back on this point in the Discussion section.

Finally, the absence of effect of the presence of the flame retardant, ATH, and of the secondary antioxidant Irganox PS802 is retrieved whatever the dose rate. Whatever the conditions, there is no interaction at the molecular level between the flame retardant and the organic part of the formulated polymers.

## 4. Discussion

The seven materials of this study can be separated into two groups, the first one containing the materials without the primary antioxidant (i.e., XLPE M1, XLPE M3(IrgPS802), XLPE M5(ATH25), and XLPE M6(ATH50)) and the second one with the materials that contain Irganox 1076 (i.e., XLPE M2(Irg1076), XLPE M4(Irg1076-IrgPS802), and XLPE M7(Irg1076-IrgPS802-ATH50)). This evidences the important effect of the presence of this primary antioxidant. 

For the first group, without primary antioxidant, the evolution the gases emission and oxygen consumption are following the equation of Decker and Mayo [47]: the higher the dose rate, the lower the oxygen consumption and, thus, consequently, the lower the quantity of defects in the irradiated material. This evolution is highlighted, for polyethylenes XLPE M1, XLPE M3(IrgPS802), XLPE M5(ATH25), and XLPE M6(ATH50) by the G_D_(H_2_) evolution; when dose increases, hydrogen emission decreases—the importance of the decrease being dependent of the dose rate. For these materials, oxygen consumption is roughly constant in the dose range of this study. This has been attributed to two competing reactions in the radical formation of: (i) the decrease in the radical formation in the unmodified polymer’s chains because of the presence of the radiation-induced defects, which act as energy sinks and (ii) the increase in the radicals formation because of the degradation of these energy sinks, which form new radicals.

Decker and Mayo [47] have shown that when an antioxidant is present in the material, the rate of oxygen consumption—and hence the quantity of defects—increases very slowly with the irradiation dose up to the exhaustion of the antioxidant and then increases equivalently to the unprotected material. This is the case of the second group, that is for materials being protected by a primary antioxidant (i.e., XLPE M2(Irg1076), XLPE M4(Irg1076-IrgPS802) and XLPE M7(Irg1076-IrgPS802-ATH50)). For these polyethylenes, the oxygen consumption radiation chemical yields are low at low dose and then increases with dose, highlighting the consumption of Irganox 1076 with dose. It can moreover be remarked on Figure 4 that at the highest dose of this study, i.e., 374 kGy, the oxygen consumption radiation chemical yield is roughly the same for the seven materials of this study (to the organic content). This observation can be explained by a total consumption of the primary antioxidant at such high dose, this hypothesis being confirmed by results of Przybytniak et al. [48]. These authors found that in the same conditions and for the same polymers, Irganox 1076 is entirely consumed at a dose around 374 kGy. Materials will thus begin to degrade at this high dose. As at low dose rate and up to 138 kGy, the evolution of the gases perfectly follows the evolution of the gases emitted and consumed at medium dose rate, it can be concluded that Irganox 1076 is not totally consumed in XLPE M2(Irg1076), XLPE M4(Irg1076-IrgPS802) and XLPE M7(Irg1076-IrgPS802-ATH50) polymers at 138 kGy when irradiated at low dose rate and that the protection of the primary antioxidant remains effective at this dose. Both doses are far higher than the ones expected in NPPs after 60 years of service.

The second observation arising from this study is that neither the flame retardant nor the secondary antioxidant has an effect on the gases emission and consumption, which means that there is no effect of these molecules at the molecular level, whatever the dose and the dose rate—in the ranges of study. In case of ATH, this result might be surprising as effects have from time to time been reported in the literature, but it should be kept in mind the distinction between the molecular scale where no effect of the ATH presence has been reported in the literature [48,49], and macroscopic scale where an pronounced effect of this flame retardant has been observed [17,18,20].

The action of thioester antioxidant is to decompose hydroperoxides, leading to the formation of alcohols on one side and of sulfoxides and sulfonic acid on the other side [44,50,51,52,53]. Hence, Irganox PS802 stabilizes the polymer by avoiding or at least reducing the in-chain oxidation mechanism, and an effect should thus be visible on the oxidized gases consumption and emission—at least at high doses where the hydroperoxides bonds are in non-negligible concentration. However, no effect of this secondary antioxidant is observed in this study. Two hypotheses can be advanced, the first one being that this kind of antioxidant is only efficient at temperatures more important than room temperature. It is in fact more generally used in thermo-oxidation ageing studies [54,55], whereas the present study is realized at moderated irradiation temperature. A second hypothesis is that the concentration of hydroperoxide is not sufficient in the material for this reaction to be effective and observable. 

## 5. Conclusions

In the context of NPP lifetime extensions from 40 to 60 years, the behavior upon irradiation under oxidative conditions of different crosslinked polyethylenes XLPE has been evaluated, as a function of the additives and fillers added in the raw material but also as a function of the irradiation conditions. Evaluation of the modifications in the materials has been realized through the quantification of emitted hydrogen, of oxygen consumption and of carbon dioxide release.

At the very beginning of the materials ageing, the additives and fillers have no effect on the hydrogen release, but presence of Irganox 1076 allows an important decrease of the oxygen consumption and of the carbon dioxide release. This confirm the important protection against oxidation given by this molecule.

When dose increases, the gases emission and oxygen consumption radiation chemical yields evolutions are explained in concordance with the pioneering results of Decker and Mayo [47]. In absence of Irganox 1076, the lower the dose rate, the higher the oxidation and the defects formation—hydrogen evolution being representative of the concentration of these defects. In presence of the primary antioxidant, the oxygen consumption increases very slowly with dose up to the exhaustion of the antioxidant. Total consumption of Irganox 1076 was determined to be roughly 374 kGy at a medium dose rate, and strictly higher than 138 kGy at a low dose rate. Both doses are far higher than the expected dose received by cables in the NPPs after 60 years of service; the good behavior at the chemical level of XLPE cables containing Irganox 1076 is here demonstrated. 

Finally, it has been evidenced that in the conditions of this study and, there is no effect of the presence of ATH—except obviously the “dilution” effect. The secondary antioxidant Irganox PS802 has been shown not to protect the polymer, even at highest dose of this study and at a temperature of 47 °C, which is a temperature equivalent to the one encountered in NPPs, at the closest of the reactor. It might be supposed that this secondary antioxidant is not necessary in the NPPs cable formulation. Investigations need to be evaluated further. 

## Figures and Tables

**Table 1:** Radiation chemical yields extrapolated at zero dose, obtained for the seven samples.

	G_0_(H_2_)	G_0_(-O_2_)	G_0_(CO_2_)
	(10^−7^ mol·J^−1^)		
XLPE M1	3.7 ± 0.3	17.9 ± 2.4	0.91 ± 0.16
XLPE M2(Irg1076)	3.4 ± 0.3	4.6 ± 0.5	0.20 ± 0.02
XLPE M3(IrgPS802)	3.3 ± 0.3	12.0 ± 1.2	0.66 ± 0.07
XLPE M4(Irg1076-IrgPS802)	3.3 ± 0.3	6.5 ± 0.7	0.29 ± 0.03
XLPE M5(ATH25)	2.9 ± 0.3	11.2 ± 1.1	0.63 ± 0.06
XLPE M6(ATH50)	2.7 ± 0.3	10.7 ± 2.1	0.67 ± 0.17
XLPE M7(Irg1076-IrgPS802-ATH50)	2.4 ± 0.3	4.6 ± 0.5	0.28 ± 0.03

## Data Availability

The data that support the findings of this study are available from the corresponding author upon reasonable request.

## References

[1] EDF, Framatome, IRSN, CEA, UJV, ARTTIC, Nexans, INCT, IZFP, VTT (2017). TeaM Cables (European Tools and Methodologies for an Efficient Ageing Management of Nuclear Power Plant Cables).

[2] Gillen T.K., Clough R.L. (1981). Occurence and implications of radiation dose-rate effects for material aging studies. Radiat. Phys. Chem..

[3] Ekelund M., Azhdar B., Hedenqvist M.S., Gedde U.W. (2008). Long-term performance of poly(vinyl chloride) cables, Part 2: Migration of plasticizer. Polym. Degrad. Stab..

[4] Ekelund M., Edin H., Gedde U.W. (2007). Long-term performance of poly(vinyl chloride) cables. Part 1: Mechanical and electrical performances. Polym. Degrad. Stab..

[5] Zaikov G.E., Gumargalieva K.Z., Pokholok T.V., Moiseev Y.V. (1998). PVC Wire Coatings: Part l-Ageing Process Dynamics. Int. J. Polym. Mater..

[6] Beneš M., Milanov N., Matuschek G., Kettrup A., Plaček V., Balek V. (2004). Thermal degradation of PVC cable insulation studied by simultaneous TG-FTIR and TG-EGA methods. J. Therm. Anal. Calorim..

[7] Gillen K.T., Assink R., Bernstein R., Celina M. (2006). Condition monitoring methods applied to degradation of chlorosulfonated polyethylene cable jacketing materials. Polym. Degrad. Stab..

[8] Gillen K.T., Bernstein R., Celina M. (2005). Non-Arrhenius behavior for oxidative degradation of chlorosulfonated polyethylene materials. Polym. Degrad. Stab..

[9] Gillen K.T., Bernstein R., Clough R.L., Celina M. (2006). Lifetime predictions for semi-crystalline cable insulation materials: I. Mechanical properties and oxygen consumption measurements on EPR materials. Polym. Degrad. Stab..

[10] Gueguen V., Audouin L., Pinel B., Verdu J. (1994). Lifetime prediction in the case of radiooxidative ageing of an ethylene/propylene rubber used for electrical insulation. Polym. Degrad. Stab..

[11] Ito M. (1981). Application of chemorheology to radiation damage of polymers. III: Synergism of heat and radiation on the chemorheology of ethylene-propylene rubber. Radiat. Phys. Chem..

[12] Lustiger A., Markham R.L. (1983). Importance of tie molecules in preventing polyethylene fracture under long-term loading conditions. Polymer.

[13] Mareş G., Ciutacu S., Budrugeac P., Chiparǎ M. (1991). Determination of the lifetime of ethylene-propylene rubber under the simultaneous action of heat and ionizing radiation. Polym. Degrad. Stab..

[14] Mopsik F.I. (1993). Radiation-induced dielectric loss in hydrocarbon polymers. J. Polym. Sci. Part B Polym. Phys..

[15] Reynolds A.B., Bell R.M., Bryson N.M.N., Doyle T.E., Hall M.B., Mason L.R., Quintric L., Terwilliger P.L. (1995). Dose-rate effects on the radiation-induced oxidation of electric cable used in nuclear power plants. Radiat. Phys. Chem..

[16] Seguchi T., Yamamoto Y. (1986). Diffusion and Solubility of Oxygen in Gamma-Ray Irradiated Polymer Insulation Materials.

[17] Azizi H., Barzin J., Morshedian J. (2007). Silane crosslinking of polyethylene: The effects of EVA, ATH and Sb_2_O_3_ on properties of the production in continuous grafting of LDPE. Express Polym. Lett..

[18] Colombani J., Sidi A., Larche J.F., Taviot-Guého C., Rivaton A. (2018). Thermooxidative degradation of crosslinked EVA/EPDM copolymers: Impact of Aluminium TriHydrate (ATH) filler incorporation. Polym. Degrad. Stab..

[19] Przybytniak G., Boguski J., Placek V., Verardi L., Fabiani D., Linde E., Gedde U.W. (2015). Inverse effect in simultaneous thermal and radiation aging of EVA insulation. Express Polym. Lett..

[20] Sidi A., Colombani J., Larché J.F., Rivaton A. (2018). Multiscale analysis of the radiooxidative degradation of EVA/EPDM composites. ATH filler and dose rate effect. Radiat. Phys. Chem..

[21] Hegazy E.-S.A., Seguchi T., Machi S. (1981). Radiation-induced oxidative degradation of poly(vinyl chloride). J. Appl. Polym. Sci..

[22] Hoffendahl C., Fontaine G., Duquesne S., Taschner F., Mezger M., Bourbigot S. (2015). The combination of aluminum trihydroxide (ATH) and melamine borate (MB) as fire retardant additives for elastomeric ethylene vinyl acetate (EVA). Polym. Degrad. Stab..

[23] Vaari J., Paajanen A. (2018). Evaluation of the reactive molecular dynamics method for Research on flame retardants: ATH-filled polyethylene. Comput. Mater. Sci..

[24] Aymes-Chodur C., Betz N., Legendre B., Yagoubi N. (2006). Structural and physico-chemical studies on modification of polypropylene and its polyphenolic antioxidant by electron beam irradiation. Polym. Degrad. Stab..

[25] Ekelund M., Fantoni P.F., Gedde U.W. (2011). Thermal ageing assessment of EPDM-chlorosulfonated polyethylene insulated cables using line resonance analysis (LIRA). Polym. Test..

[26] Moisan J.Y., Lever R. (1982). Diffusion des additifs du polyethylene—V: Influence sur le vieillissement du polymere. Eur. Polym. J..

[27] Celette N., Stevenson I., Davenas J., David L., Vigier G. (2001). Relaxation behaviour of radiochemically aged EPDM elastomers. Nucl. Instrum. Methods Phys. Res. Sect. B Beam Interact. Mater. At..

[28] Celina M.C. (2013). Review of polymer oxidation and its relationship with materials performance and lifetime prediction. Polym. Degrad. Stab..

[29] Howard J.B. (1973). DTA for control of stability in polyolefin wire and cable compounds. Polym. Eng. Sci..

[30] Bartoníček B., Hnát V., Plaček V. (1998). Life-assessment technique for nuclear power plant cables. Radiat. Phys. Chem..

[31] Xu A., Roland S., Colin X. (2020). Physico-chemical characterization of the blooming of Irganox 1076^®^ antioxidant onto the surface of a silane-crosslinked polyethylene. Polym. Degrad. Stab..

[32] Ferry M., Pellizzi E., Boughattas I., Fromentin E., Dauvois V., de Combarieu G., Coignet P., Cochin F., Ngono-Ravache Y., Balanzat E. (2016). Effect of cumulated dose on hydrogen emission from polyethylene irradiated under oxidative atmosphere using gamma rays and ion beams. Radiat. Phys. Chem..

[33] Fromentin E., Aymes-Chodur C., Doizi D., Cornaton M., Miserque F., Cochin F., Ferry M. (2017). On the radio-oxidation, at high doses, of an industrial polyesterurethane and its pure resin. Polym. Degrad. Stab..

[34] Lebeau D., Beuvier L., Cornaton M., Miserque F., Tabarant M., Esnouf S., Ferry M. (2015). Aging of magnesium stearate under high doses gamma irradiation and oxidative conditions. J. Nucl. Mater..

[35] Ferry M., Dannoux-Papin A., Dély N., Legand S., Durand D., Roujou J.L., Lamouroux C., Dauvois V., Coignet P., Cochin F. (2014). Chemical composition effects of methylene containing polymers on gas emission under γ-irradiation. Nucl. Instrum. Methods Phys. Res. Sect. B Beam Interact. Mater. At..

[36] Ferry M., Bessy E., Harris H., Lutz P.J., Ramillon J.M., Ngono-Ravache Y., Balanzat E. (2013). Aliphatic/Aromatic Systems under Irradiation: Influence of the Irradiation Temperature and of the Molecular Organization. J. Phys. Chem. B.

[37] Manion J.P., Burton M. (1952). Radiolysis of Hydrocarbon Mixtures. J. Phys. Chem..

[38] Norrish R.G.W., Bamford C.H. (1937). Photo-decomposition of Aldehydes and Ketones. Nature.

[39] Schoepfle C.S., Fellows C.H. (1931). Gaseous Products from Action of Cathode Rays on Hydrocarbons. Ind. Eng. Chem..

[40] Bolland J.L., Gee G. (1946). Kinetic studies in the chemistry of rubber and related materials. II. The kinetics of oxidation of unconjugated olefins. Trans. Faraday Soc..

[41] Arakawa K., Seguchi T., Watanabe Y., Hayakawa N. (1982). Radiation-induced oxidation of polyethylene, ethylene-butene copolymer, and ethylene-propylene copolymer. J. Polym. Sci. Polym. Chem. Ed..

[42] Matsuo H., Dole M. (1959). Irradiation of Polyethylene. IV. Oxidation Effects. J. Phys. Chem..

[43] Rabek J.F. (1996). Photodegradation of Polymers, Physical Characteristics and Applications.

[44] Gijsman P., Kutz M. (2018). Chapter 18—Polymer Stabilization. Handbook of Environmental Degradation of Materials.

[45] Ventura A., Ngono-Ravache Y., Marie H., Levavasseur-Marie D., Legay R., Dauvois V., Chenal T., Visseaux M., Balanzat E. (2016). Hydrogen emission and macromolecular radiation-induced defects in polyethylene irradiated under an inert atmosphere: The role of energy transfers toward trans-vinylene unsaturations. J. Phys. Chem. B.

[46] Seguchi T. (2001). Mechanisms and kinetics of hydrogen yield from polymers by irradiation. Nucl. Instrum. Methods Phys. Res. Sect. B Beam Interact. Mater. At..

[47] Decker C., Mayo F.R. (1973). Aging and degradation of polyolefins. II. γ-initiated oxidations of atactic polypropylene. J. Polym. Sci. Polym. Chem. Ed..

[48] Przybytniak G., Sadło J., Walo M., Wróbel N., Žák P. (2020). Comparison of radical processes in non-aged and radiation-aged polyethylene unprotected or protected by antioxidants. Mater. Today Commun..

[49] Maléchaux A., Colombani J., Amat S., Marque S.R., Dupuy N. (2020). Influence of gamma irradiation on electric cables: Study of additive effects by mid infrared spectroscopy. Polymers.

[50] Armstrong C., Husbands M.J., Scott G. (1979). Mechanisms of antioxidant action: Antioxidant-active products formed from the dialkyl thiodipropionate esters. Eur. Polym. J..

[51] Bateman L., Hargrave K.R., Rideal E.K. (1954). Oxidation of organic sulphides. I. Interaction of cyclohexyl methyl sulphide with hydroperoxides in alcohols. Proc. R. Soc. London. Ser. A Math. Phys. Sci..

[52] Bateman L., Hargrave K.R., Rideal E.K. (1954). Oxidation of organic sulphides-II. Interaction of cyclohexyl methyl sulphide with hydroperoxides in hydrocarbons. Proc. R. Soc. London. Ser. A Math. Phys. Sci..

[53] Richaud E., Monchy-Leroy C., Colin X., Audouin L., Verdu J. (2009). Kinetic modelling of stabilization coupled with stabilizer loss by evaporation. Case of dithioester stabilized polyethylene. Polym. Degrad. Stab..

[54] Verdu J., Rychly J., Audouin L. (2003). Synergism between polymer antioxidants—kinetic modelling. Polym. Degrad. Stab..

[55] Xu A., Roland S., Colin X. (2020). Thermal ageing of a silane-crosslinked polyethylene stabilised with a thiodipropionate antioxidant. Polym. Degrad. Stab..

